# Challenges in the use of the CytoSorb^®^ adsorber in an intensive care patient with liver dysfunction of unknown origin: a case report

**DOI:** 10.1186/s13256-025-05503-9

**Published:** 2025-09-08

**Authors:** Caroline Gräfe, Michael Paal, Michael Irlbeck, Uwe Liebchen, Christina Scharf, Helen Graf

**Affiliations:** 1 Department of Anesthesiology, LMU University Hospital Munich LMU, Marchioninistrasse 15, 81377 Munich, Germany; 2 Institute of Laboratory Medicine, LMU University Hospital LMU, Marchioninistr. 15, 81377 Munich, Germany

**Keywords:** CytoSorb^®^, Bile acids, Liver, Vancomycin, Intensive care medicine

## Abstract

**Background:**

The treatment of critically ill patients in intensive care units is becoming increasingly complex. For example, organ transplants are regularly carried out, the recipients are seriously ill, and the postoperative course can be complicated. This is why organ replacement and hemadsorption procedures are becoming increasingly important. Adsorption processes are used for specific indications, such as hyperinflammation, hepatic dysfunction, or rhabdomyolysis, in critically ill patients. Nevertheless, there is still a knowledge gap in terms of safety, interactions, and application. This case report provides an example of the thought process that went into deciding whether to apply an adsorption process, as well as other necessary treatment modifications resulting from the application.

**Case presentation:**

We present the case of a 26-year-old Italian man with a complicated postoperative period after lung transplant with the need of organ support systems and hemadsorption therapy. Besides operative difficulties of the transplant and massive bleeding, the patient developed a severe liver failure of unknown origin with highly elevated bile acids, which indicated the use of the CytoSorb^®^ cytokine adsorber. Since there are indications that undesired drug elimination may occur and that saturation kinetics have not yet been comprehensively investigated, there were a number of aspects to consider during application. For example, the patient received additional vancomycin dosing and vancomycin blood levels were monitored. Further, to avoid premature saturation of the adsorber, the cartridge was changed every 8 hours. These adjustments resulted in a continuous reduction in bile acids while maintaining stable vancomycin blood levels, which is critical in immunosuppressed patients.

**Conclusion:**

The report focuses on two main aspects regarding a safe and sufficient usage of CytoSorb^®^ in the intensive care medicine. First, shortened changing periods increase the elimination rate of the adsorber, which is quickly saturated by bigger molecules, for example, bile acids. Second, additional vancomycin dosing during CytoSorb^®^ application avoids under dosing since CytoSorb^®^ eliminates vancomycin.

## Introduction

Caring for patients before and after transplantation is part of everyday life for many intensive care units (ICUs) today. However, the care of this group of patients is inconceivable without organ replacement procedures, which make it possible to bridge the gap until the new organs are fully functional. These include, for example, renal replacement therapy (RRT) or extracorporeal membrane oxygenation (ECMO). Additionally, sometimes the transplanted organ is not the only organ that requires artificial support. In the presented case the patient underwent a lung transplant with the need of bridge-to-transplant ECMO therapy. After a challenging lung transplant his lung function was significantly improved but he developed a liver dysfunction of unknown origin. In a retrospective study 1406 adult lung transplant recipients and the risk of liver dysfunction were evaluated. A total of 8.3% developed a liver dysfunction whereby pre-known liver disease was the most important risk factor [1]. Liver dysfunction is a severe risk factor for mortality and morbidity not only in transplant medicine. One reason for the high mortality risk in patients with liver dysfunction is that, despite our efforts, we cannot yet fully replace liver function. Adsorption processes, such as the cytokine adsorber CytoSorb^®^ (CS), are used for liver support therapy in critically ill patients. It contains polymeric beads with a total surface area of up to 45,000 m^2^ that can unselectively bind hydrophobic molecules < 60 kDa [2]. It can be used for the elimination of bilirubin and bile acids in patients with liver dysfunction, cytokine reduction during hyperinflammatory conditions, and myoglobin removal in patients with rhabdomyolysis. In addition, the adsorber can be used to eliminate anticoagulants such as ticagrelor and rivaroxaban [2–6]. The adsorber can be used in hemodialysis or ECMO circuits or as a stand-alone application. Besides the desirable elimination of cytokines, bilirubin, myoglobin and anticoagulants, there are also an increasing number of studies showing the undesirable elimination of certain anti-infectives [7], for example. The exact elimination processes have not yet been fully investigated and are the subject of current research. Therefore, the use of CS in critically ill patients should be carefully considered and its effects on other treatments should be considered and corrected if necessary.

## Case presentation

We present the case of a 26-year-old Italian man suffering from an interferon-gamma-receptor defect. Thus, he underwent several bone marrow transplants in his youth. Unfortunately, he developed bronchiolitis obliterans owing to graft-versus-host disease (GvHD). On account of the emphysematous changes in the pulmonary framework, his pulmonary function decreased, and he was listed for a lung transplant. Prior to transplantation a veno-venous-ECMO had to be established as bridge to transplant therapy and an appropriate organ was allocated after 57 days with vv-ECMO support. The transplantation was demanding. A large blood loss and subsequent coagulation failure required mass transfusion and a thoracic packing after lung transplantation. The patient was routinely transmitted to the ICU still requiring vv-ECMO therapy, mechanical ventilation and catecholamine therapy. He was sedated with sufentanil, propofol and midazolam (Richmond Agitation Sedation Scale (RASS) -5). The immunosuppressive therapy was started directly with prednisolone (starting with 60 mg/day) and tacrolimus (starting with continuously 40 μg/hour intravenous) according to internal standard. During the first postoperative day, the patient’s condition deteriorated: The required catecholamine doses increased to 0.44 µg/kg/minute and the ventilation was increasingly restricted (minute volume 1.7 l/minute, expiratory tidal volume 64 ml). A rapidly increasing lactate and acute kidney injury (AKI) necessitated the initiation of continuous renal replacement therapy (RRT). Furthermore, liver dysfunction with hyperbilirubinemia (2.9 mg/dL, threshold value 1.2 mg/dL) and elevated transaminases (AST 3547 U/L, ALT 2438 U/L) was detected. The CT-scan revealed suspected nonocclusive mesenteric ischemia and active bleeding out of a pulmonary vein. In consequence, the patient underwent a revision surgery including a laparotomy without evidence of mesenteric ischemia.

The liver failure aggravated in the following days without a concrete reason to be found. The ultrasound examination did not reveal any pathological findings and the constellation of laboratory values did not indicate a clear cause. Anti-human *t*-lymphocyte immunoglobulin (ATG) was prescribed in case of an immunological reason considering the patient’s medical history. In addition, the attending physicians decided to use the adsorber CytoSorb^®^ to eliminate the increasing bile acids (228 μmol/L before CS start, threshold value < 10 μmol/L) from the patient’s blood as an additive therapy strategy. They changed the CS cartridge every eight hours and used five cartridges in total. During CS application, the bile acids concentrations were regularly monitored and CS therapy was continued in accordance to the clinical appearance and the laboratory measurements. Bile acid concentrations before cartridge changes were 134 μmol/L, 104 μmol/L and 97.4 μmol/L. Finally, the therapy was ended when the bile acid concentration stabilized and normalized on day 46. There were no side effects directly related to CS, particularly no further bleeding. The patient received a perioperative therapy with meropenem (250 mg/h) according to internal standard. Furthermore, vancomycin was administered as continuous infusion (60 mg/h) during ECMO therapy to avoid cannula infections.

At the beginning of each CS application, 500 mg vancomycin was administered as an additional dose over two hours. Regular therapeutic drug monitoring (TDM) of vancomycin was performed, resulting in vancomycin concentrations over the lower target level of 20 mg/L (day 5 after lung transplant 24.5 mg/L, day 6 27.4 mg/L, day 7 (end of CS) 26.6 mg/L, day 8 21.1 mg/L).

**Fig. 1:**
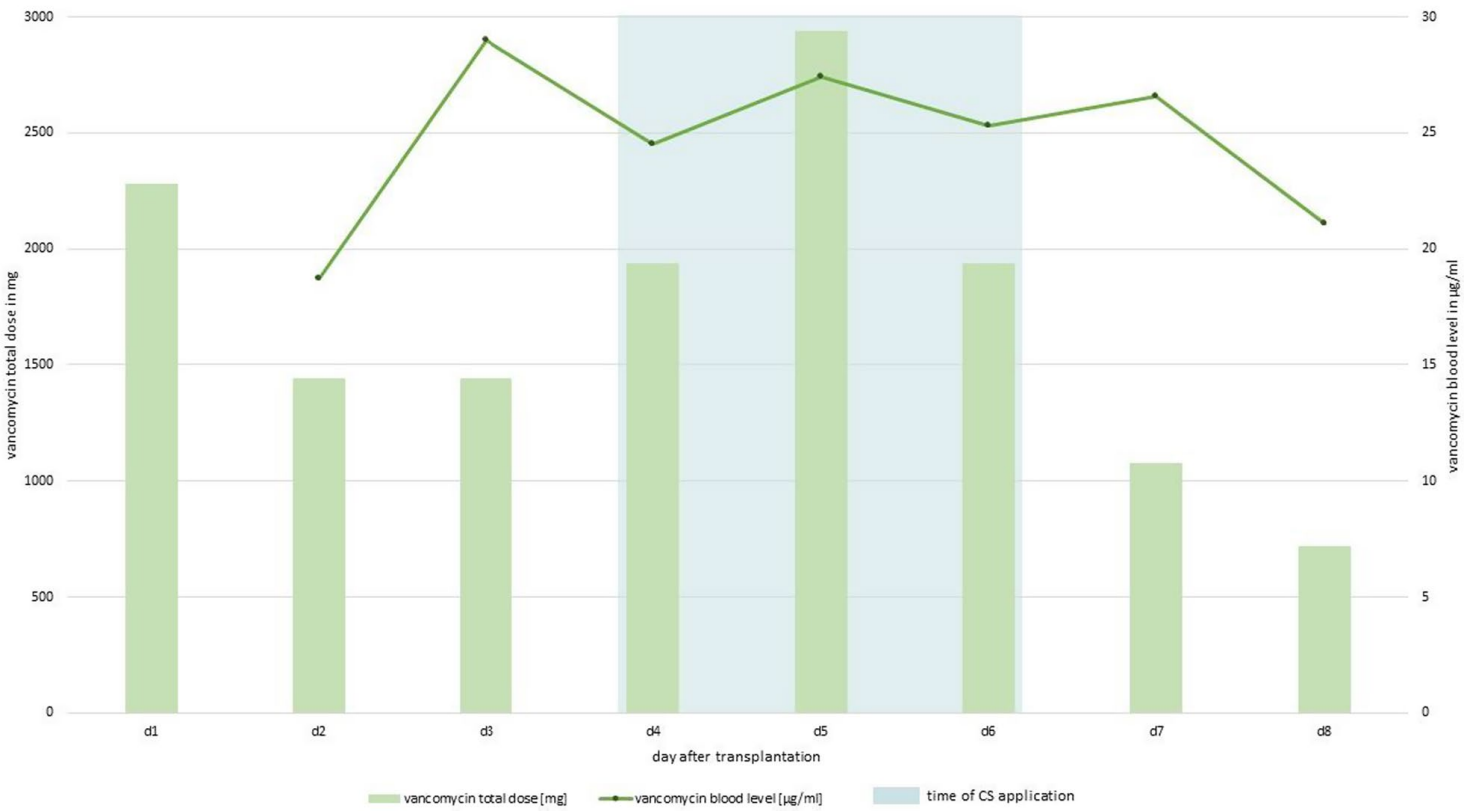
Vancomycin concentrations and daily doses in the first 8 days after lung transplant. D1-d8 mark the days after lung transplantation. The green line graph shows the vancomycin blood levels and the green bars show the total dose of vancomycin for each day. The blue shaded areas marks the time of CytoSorb^®^ application (a total of five adsorbers in a row)

Figure 1 shows the vancomycin levels and the total daily dose resulting from the additional doses and continuous infusion.

**Fig. 2:**
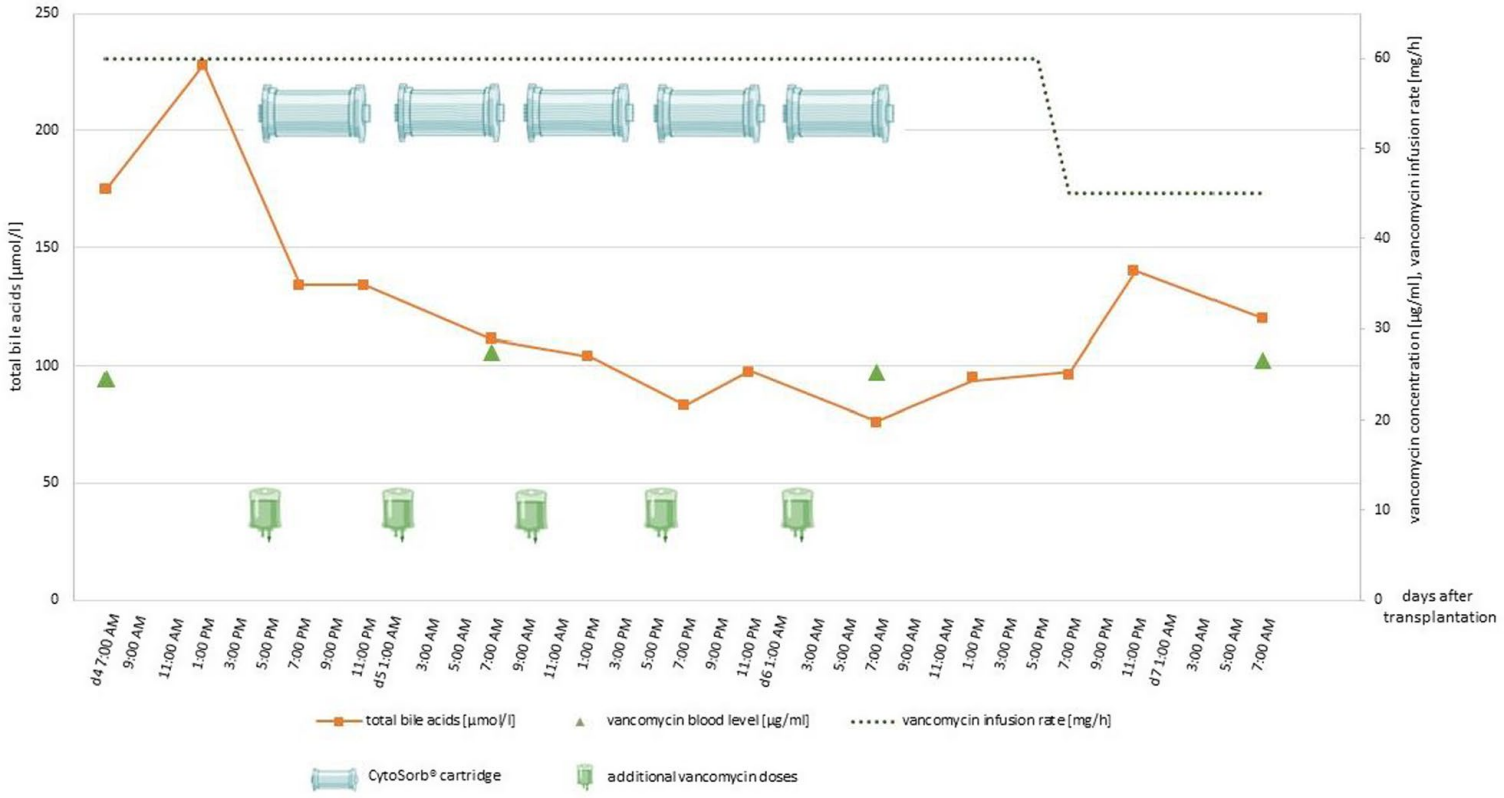
Total bile acids, vancomycin blood levels as well as additional and continuous vancomycin doses during CytoSorb^®^ application. The orange line graph shows the total bile acid serum concentration during CytoSorb^®^ application. The green triangles mark the vancomycin blood levels and the dark green dotted line the vancomycin infusion rate. The green infusion bags symbolize the additional vancomycin doses of 500 mg over 2 hours and the cartridges symbolize the CytoSorb^®^ application

Figure 2 provides detailed information about the total bile acids and vancomycin blood levels as well as the vancomycin infusion rate and additional vancomycin doses during the hours of CS application.

In the following days, the patient’s condition improved and vv-ECMO therapy could be terminated ten days after the lung transplantation. Liver (day of ICU discharge: bilirubin 0.8 mg/dl, bile acids 2.3 μmol/L, AST 18 U/L, ALT 22 U/L) and renal (day of ICU discharge: creatinine 0.9 mg/dL) function fully recovered and dialysis was permanently ended on day 33 after transplantation. The patient was transferred to the normal ward on day 50 after transplantation in a good condition and a sufficient graft function. Written informed consent was obtained from the patient for publication of the case.

## Discussion

The presented case describes the complicated course of a lung transplantation in a young man with bronchiolitis obliterans due to a GvHD after several bone marrow transplants because of an interferon-gamma-receptor defect. The case itself has many interesting aspects, but we would like to focus on the application of the adsorber CS in this case report. In general, it should be noted that there is currently no evidence that CS therapy positively affects patient mortality, regardless of the area of application. Therefore, CS therapy is not generally recommended in intensive care medicine [8]. Unfortunately, there are also no general protocols for CS use and so far, only relatively few recommendations in the literature. Klinkmann et al. [9] summarized the current recommendations in 2023 and also point out that CS use should be an individual decision. Their recommendations also include early use of CS, no use with lactate levels ≥ 6 mmol/L or platelets < 100 GPT/L and regular drug monitoring if possible.

The first point we want to highlight about our case is that CS was used to indicate liver dysfunction of unknown origin with elevated bile acids. Greimel et al. recently showed that CS eliminates protective and toxic bile acids from the patients’ blood but showed a fast saturation of the cartridge after about six hours [10]. In accordance to these findings, the CS cartridges used in our case were changed after 8 hours and not after 12–24 hours, which is the usual period stated by the manufacturer. A continuous decline of total bile acid concentration during CS therapy was observed in our patient.

In a retrospective analysis, Hyzny et al. evaluated 1406 adult lung transplant recipients and the risk of liver dysfunction [1]. A total of 8.3% developed a liver dysfunction whereby pre-known liver disease was the most important risk factor. Additionally, they showed that postoperative liver dysfunction is associated with higher mortality and morbidity. Weig et al. retrospectively evaluated patients listed for lung transplant and vv-ECMO as bridge to transplant. They showed that non-surviving patients after lung transplant had significantly more frequently liver dysfunction with hyperbilirubinemia during the bridging period [11]. Even though our patient had no pre-known liver disease, these results underline the relevance of this clinical picture. As reason for his liver dysfunction remains unclear, the causative reason for the organ recovery cannot be named. However, CS is an additional and supportive therapeutic option and the elimination of bile acids might be helpful since liver function recovered quickly after CS therapy.

The second aspect we wanted to highlight is the performance of anti-infective therapy during CS application. Scharf et al. showed that CS significantly eliminates vancomycin in a clinically relevant amount [7]. They suggested an additional dose of 500 mg vancomycin according to a pharmacokinetic model. Even though the patient received up to twice of the normal daily dose due to the additional doses, vancomycin levels did not significantly rise above target. This confirms the adsorption of vancomycin through CS, which is crucial to know for a safer use of CS. Since, according to Liebchen et al., a relevant removal of meropenem by CS was not expected additional dosing was not performed [12]. Patients in the ICU receive a variety of medications, so it would be useful to know how CS treatment affects them all. Unfortunately, due to non-selective absorption, only limited predictions can be made about what the effect might look like. However, some studies have already addressed this topic, but it should be noted that most of the results here are for the two drug groups: anti-infectives and anticoagulants. An article by Scheier et al. [13] provides an excellent summary of the known effects of CS on drug levels. Currently, there is no evidence that immunosuppressive agents [14] or sedatives are affected by CS. To the authors’ best knowledge, this is the first case report, which evaluates this pragmatic approach. The vancomycin levels during CS application with the suggested additional doses were stable over time. Anti-infective therapy is an important component of intensive care medicine and is particularly important for immunosuppressed patients after organ transplantation. Therefore, the knowledge of effects on the concentrations of anti-infectives is important and potentially lifesaving [15]. This case report may influence other physicians to watch out carefully for the effect of CS on drug levels and encourage further studies in this area.

## Conclusion

This case report outlines the key considerations for practitioners when using CS in intensive care to treat their patients effectively and safely. The focus is on two main aspects. Firstly, owing to saturation of the adsorber by larger molecules such as bile acids and myoglobin, shortening the changing period to 8 hours improves the elimination rate and leads to more effective therapy. Secondly, additional vancomycin dosing during CS application prevents underdosing, as CS eliminates vancomycin. Treating critically ill patients is complex, and using a nonselective adsorber sometimes requires careful consideration to ensure it is used effectively.

## Data Availability

The data that support the findings of this study are available from the corresponding author upon reasonable request.

## Abbreviations

AKI: Acute kidney injury
ATG: Anti-human t-lymphocyte immunoglobulin
CS: CytoSorb^®^
ECMO: Extracorporeal membrane oxygenation
GvHD: Graft versus Host disease
ICU: Intensive care unit
RRT: Renal replacement therapy
